# Conductometric Flexible CuO-Based Sweat-Loss Monitoring Sensor for Future Wearable Technology in Healthcare

**DOI:** 10.1021/acsomega.3c05278

**Published:** 2023-11-01

**Authors:** Rasit Aydin, Osman Kahveci, Abdullah Akkaya, Bünyamin Şahin, Enise Ayyıldız

**Affiliations:** † Department of Physics, Faculty of Sciences, Selçuk University, Konya 42130, Turkey; ‡ Department of Physics, Faculty of Sciences, Erciyes University, Kayseri 38039, Turkey; § Mucur Technical Vocational Schools, Tech. Prog. Department, Kırşehir Ahi Evran University, Kırşehir 40100, Turkey; ∥ Department of Basic Sciences, Faculty of Engineering, Necmettin Erbakan University, Konya 42090, Turkey

## Abstract

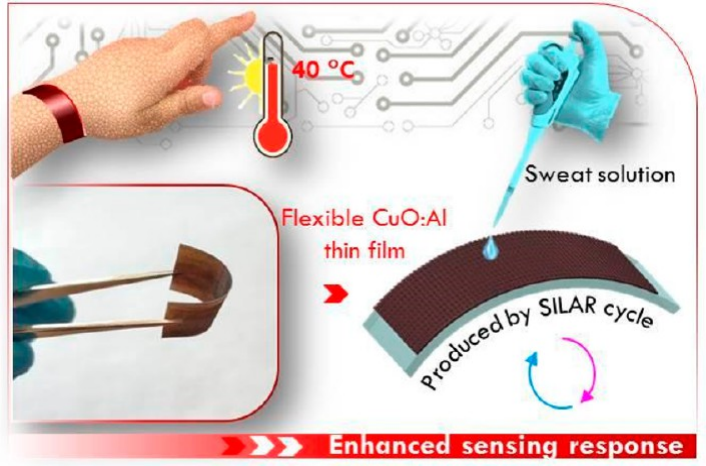

Healthcare tracking supports early diagnosis of illnesses, real-time tracking of the impacts of therapy and treatment, and tracking of cases of human health. Based on this, real-time tracking of sweat loss provides an easy, convenient, and noninvasive approach to the early diagnosis of physical illness in individuals. To date, copper oxide (CuO) as a nanostructured semiconductor metal-oxide is regarded as a hopefully p-type sensing material. The corresponding sweat rate monitoring systems were fabricated using a repeatable and cost-effective SILAR system using a cellulose acetate-based organic substrate. To provide a practical application, we investigated the working efficiency of Al:CuO under room conditions since most clinical and healthcare industries operate under ambient temperature. Fabricated flexible devices immediately respond to the implementation of the sweat solution and reach a steady-state value in a short time. It can be obtained from experimental results that the sweat-loss monitoring performances of CuO-based devices can be enhanced by employing Al-doping. The increment in the sensing efficacy was interpreted in terms of structural and morphological characterization and electrical data. Our designed flexible thin film-based system can be used in conjunction with a customized wearable, healthcare industry integrated, low-cost fabrication source.

## 1 Introduction

Wearable physical sensing equipment tracks different physiological parameters in real time, such as heart rate, sweat loss, blood pressure and temperature, and oxygen saturation.^1^ Sweat is a biological liquid sample secreted by the sweat glands of individuals and spread throughout the entire body. As a biological fluid, sweat contains electrolytes and metabolites, such as urine, blood, and saliva. The main compositions of sweat are primarily water and ions such as sodium (Na^+^), potassium (K^+^), and chloride (Cl^+^).^2^ The wearable sweat tracking device allows continuous, real-time, noninvasive determination of electrolytes, procuring insight into individual body condition at the molecular level, and has received growing relevance for their hopeful implementations in individualized health tracking.^3^ For example, changes in the electrolyte concentration in human sweat can be used as a biomarker to track dehydration during exercise in warm or hot conditions, which may be a significant implication for the water intake of athletes.^4^ In this view, immense struggles have been conducted to detect and collect sweat by implicating the direct connection of devices.^5^

Metal oxide semiconductor (MOs) materials have created a remarkable demand for their use in wearable technology applications due to many features such as being cheap and simple production methods, having an adjustable forbidden band gap, and having a large surface area.^6^ For this reason, they have been in-demand materials in the production of human-based flexible/wearable sensors and smart devices as well as chemical pollution (environmental) and gas leak detectors.^2a,6b,7^ In recent years, sensors built on flexible substrates for wearable technology and health monitoring monitors containing CuO, ZnO, SnO_2_, NiO, and WO_3_ MOs have been widely used.^8^ Especially, CuO is a promising candidate because of its high sensitivity, response/recovery speed, excellent stability, and cost-effective production with simple fabrication processes such as Successive Ionic Layer Adsorption and Reaction (SILAR).^2a,7a,7c^ As one of the extensive nanostructured metal oxide materials, CuO is also commonly utilized in the field of different sensor applications.^9^ In recent years, extensive research on copper oxide-based sweat sensors has been observed in the literature. Ibarlucea et al.^10^ used a flexible alginate/CuO modified electrode for electrochemical detection of ascorbic acid in artificial sweat. Nafady et al.^11^ utilized a sensor consisting of CuO nanoparticles embedded in a conductive PANI framework for the periodic detection of alcohol in sweat. Traditional sensor manufacturing techniques rely on rigid substrates due to their high manufacturing temperature, but flexible and stretchable substrates have been reported recently. These substrates are usually flexible polymeric compounds such as polyethylene terephthalate (PET), cellulose acetate (CA), and Teflon.^2a,7c,7d^ Besides all of this, the sensing properties of MO materials can also be altered by the addition of dopants. Doping can be used to change the basic properties of sensor formation, such as conductivity, morphology, surface area, and sensing ability.^2b,4a,7a,12^ In this study, aluminum (Al) was used as a doping element due to its abundant abundance, low price, low resistance, low ionization energy, high transparency, small ion radius, and low toxicity.^13^

Investigation in the area of real-time sweat sensing has exhibited a sharp upward trend in the past decade. To the best of our knowledge, Al-doped flexible CuO films produced by the SILAR method have not been previously reported in the literature. This study focuses on the potential use of flexible CuO films as sweat sensing materials for future wearable technology. Therefore, this is the first study to evaluate the material characterization and sweat-sensing capabilities of nanostructured flexible CuO films synthesized by the SILAR method depending on the Al doping contents. In this research, nanostructured flexible Al:CuO film-based devices have been evolved to monitor users’ health conditions and track the levels of electrolytes in sweat in hot and humid climates. Sweat solutions of four different contents were equipped to examine the fabricated device performance. Our conductometric experiment consequences were promising, and we hope low-cost SILAR-produced flexible copper oxide-based nanoscale thin films can be used as standalone sensing materials for real-time sweat loss tracking.

## 2 Experimental Details

Bare and Al-doped CuO films at different concentrations (1.0% and 2.0%) were grown on cellulose acetate (CA) substrates using the SILAR method. Cupric chloride dihydrate (Cl_2_CuH_4_O_2_, Merck, ≥99.0%) for the Cu^2+^ ion source and aluminum nitrate nonahydrate (AlH_18_N_3_O_18_, Sigma-Aldrich, ≥99.0%) for the Al^3+^ ion source were used in the experiments. For the synthesis of bare CuO films, 01 M Cu^2+^ ion solution was prepared using Cl_2_CuH_4_O_2_ salt in 100 mL of distilled water (18 MΩcm). The solution was then stirred in a magnetic stirrer at room temperature for a few minutes to obtain a transparent color. After the solutions were mixed, the pH of the solution was adjusted to approximately 10.0 by adding aqueous ammonia (NH_4_OH, Sigma-Aldrich). The solutions were heated to 75 °C and kept at this temperature during the growth of CuO films. CA substrates were sequentially immersed in the solution, hot water, solution, and hot water for 20 s, respectively. Thus, the SILAR cycle process was performed. Ten SILAR cycles were performed in this way to produce CuO films. To produce Al-doped CuO films, the solution was prepared by adding 1.0 and 2.0% AlH_18_N_3_O_18_ to the Cu^2+^ solution, respectively. Al-doped CuO films were grown on a CA substrate by applying the above time and cycle values.

Flexible thin films’ surface morphologies, mapping, and chemical compositions were characterized by Field Emission Scanning Electron Microscopy (FE-SEM) (Zeiss Gemini 500) and Energy Dissipative Spectroscopy (FE-SEM-EDX) (EDAX Inc.). The surface morphology of the films, such as shape parameters and roughness, was studied by using a Negra solaris atomic force microscope (AFM, NT-MDT). A D8 advance X-ray diffractometer (Bruker, operating at 30 mA and 45 kV) using Cu Kα radiation (λ = 0.15405 nm) was used to explain the crystal phase structure of CuO films. The thickness of the produced films was found using a NanoMap 500LS 3D profilometer (AEP technology). Fourier transform infrared (FT-IR) spectroscopy of the CuO films was obtained by using a VERTEX70 FT-IR spectrometer (BRUKER). The contact angle of water on the bare and Al-doped CuO films was measured using an OCA 50Micro instrument (DATAPHYSICS). The *I*–*t* (current–time) characteristics of all films were investigated using a 6487 ps (Keithley) under room conditions.

## 3 Results and Discussion

**Figure 1:**
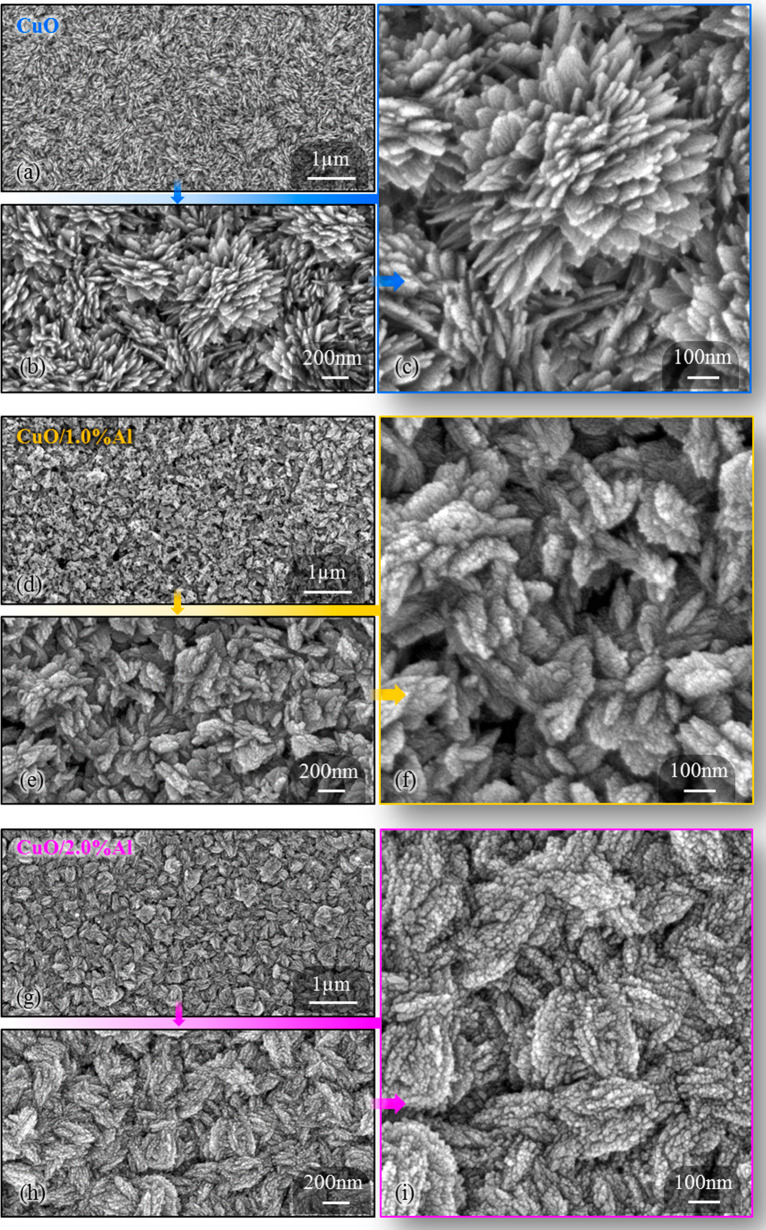
Magnified and optimum size FE-SEM images of flexible thin films. Bare CuO sample’s images are in (a) 1 μm, (b) 200 nm, and (c) 100 nm scales. CuO/1.0% Al sample’s images are in (d) 1 μm, (e) 200 nm, and (f) 100 nm scales. CuO/2.0% Al sample’s images are in (g) 1 μm, (h) 200 nm, and (i) 100 nm scales. The relatively flat plated flower-like patterned CuO structures are transformed into nanodot structures with less sharp edges by Al-doping.

The FE-SEM images of thin films prepared on flexible substrates are given in Figure 1 in detail. The morphological structure of the bare CuO film is quite different from that of the Al-doped films. Figure 1a–c shows the surface images of bare CuO thin film at different scales. The structure of the bare CuO film consisted mostly of flat-like plated flower patterns and sharp-edged structures. Figure 1d–f shows the surface images of the CuO/1.0% Al thin film. The final images in Figure 1g–i show the surface images of the CuO/2.0% Al thin film. In Al-doped films, on the other hand, fuller soft-edged structures stand out. Especially in the structure of CuO/2.0% Al film with 2.0% Al-doped, nanosized dot structures created roughness on the surface. The morphological change in the film structure by doping CuO films was quite compatible with the experimental work in the literature, the schematic drawing of which is also given.^14^

Important factors such as nucleation and crystal growth rate play an active role in the formation of crystal structure.^15^ In addition, the distinctive electronegativity of the metal doped in the oxidation process (Al in this study) may be effective in the morphology change.^16^ The electronegativities of Cu and Al are 2.00 and 1.61, respectively. The substitution of Cu with Al dopant ions causes an increase in the electron concentration and oxygen vacancies due to the difference in electronegativity between the dopant (Al) and the host elements (Cu and O). The different electronegativities of the dopants affect the the free surfaces and growth mechanism of CuO.^17^ Al doping provided morphologically significant changes in the flexible CuO film. FE-SEM images demonstrated these changes. These morphological changes may have different effects on film properties, such as film thickness and roughness. In connection with these features, the sensing ability of films can differentiate their properties. It has been reported that the morphological change strengthens the cohesion effect and plays a role in the change of the contact angle.^18^

**Figure 2:**
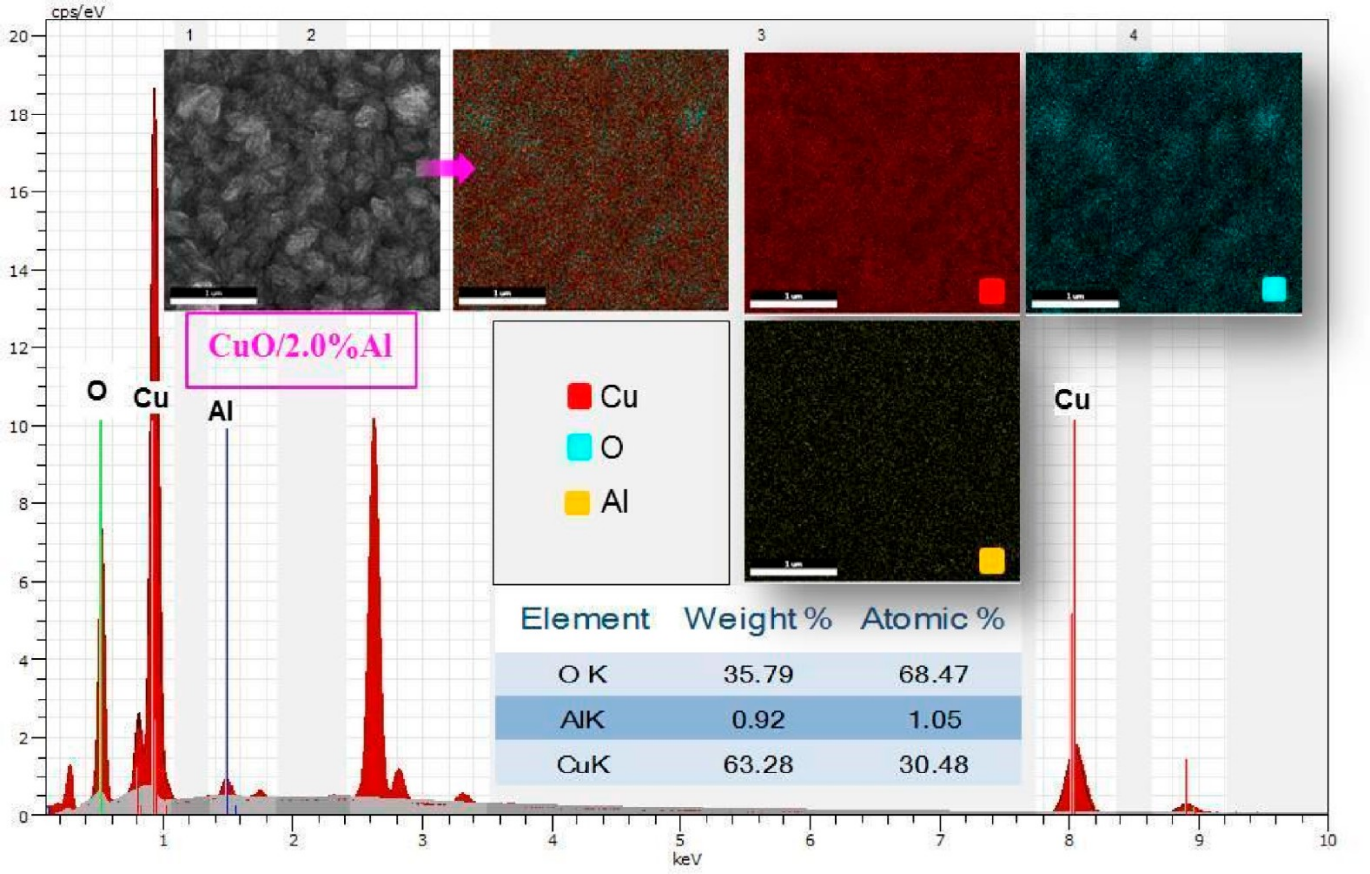
Results of EDX analysis and mapping for 2.0% Al-doped CuO film and distribution table of elements by weight and atomic percentage embedded in the figure.

The results of the EDX analysis and mapping for the 2.0% Al-doped CuO film can be seen in Figure 2. The distribution table of the elements by weight and atomic percentage is given as embedded in Figure 2. Before FE-SEM analysis, a Au coating was applied to all flexible films to provide better conductivity. As can be seen from the EDX peak graph, the peaks that may come from the flexible substrate and coating are neglected, and optimized results are given. Mapping is visualized by showing Cu in red, O in cyan, and Al in yellow. Although mapping images show that Cu and O elements are locally concentrated in clusters containing nanostructures, they are homogeneously distributed throughout the film. The Al element exhibits a homogeneous distribution in the whole structure. As a result, the existence of all of the elements in the film structure was confirmed.

**Figure 3:**
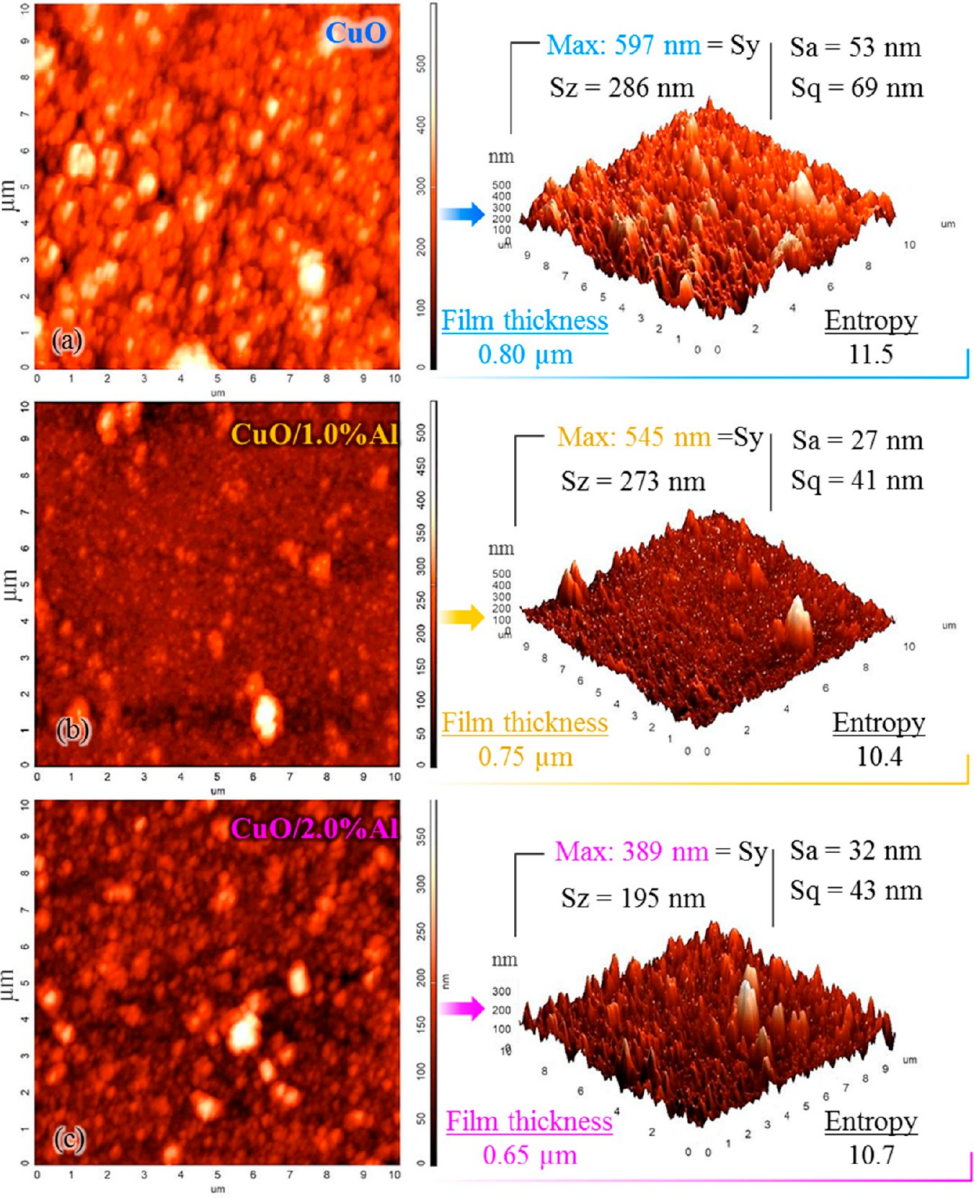
2D and 3D topographic AFM images and characteristic parameters of flexible (a) bare CuO, (b) CuO/1.0% Al, and (c) CuO/2.0% Al thin films.

Atomic force microscopy (AFM) examinations were carried out to examine the surface morphological features in detail and thus to understand the film. AFM results were also used as a supporting method to investigate how Al doping affects the functional performance of a flexible CuO film. Topographic images were made in a 10 × 10 μm scanning area. Imaging results in 2D and 3D are given in Figure 3. The characteristic parameters obtained from the film topographies are also seen in Figure 3. Figure 3a,b, and c shows the results of bare CuO, CuO/1.0% Al, and CuO/2.0% Al thin films, respectively.

The maximum scale value shown in the 3D images decreases as the Al doping rate increases. This means that the surface protrusions decrease as the doping ratio increases. Peak-to-peak (Sy) results also show this situation. Similarly, the ten-point height (Sz) results also decrease as the doping ratio increases. The contrast difference in the 2D images can explain this situation. Both the axis scale of the bare CuO film is larger and the light-colored regions are more conspicuous. The average roughness (Sa) and root-mean-square (Sq) values of the surfaces are lower for Al-doped films but slightly higher for 2.0% Al-doped CuO/2.0% Al film than for 1.0% Al-doped CuO/1.0% Al film. This may be due to the excess of dot structures detected by FE-SEM images (please remember that in FE-SEM results dot structures were observed more intensely in 2.0% Al doped film morphology). This situation seems to be similar to the structural entropy. The results are also in agreement with the CuO thin film scientific studies in the literature. For example, the Sa value of the surfaces varies between 0.5 and 76 nm in scientific studies on CuO thin films.^19^ In this study, it was determined as a minimum of 27 nm and maximum of 53 nm.

The profilometer measurement method, which is often preferred in thin film studies,^20^ was used for the measurement of film thicknesses. The profilometry results of the films with the same SILAR cycle show that the film thickness decreases as the Al doping ratio increases. The results show that Al doping of CuO films causes significant changes in surface morphology and structural properties. These results support the transformation of the CuO film structure consisting of relatively flat plates with a flower-like pattern into a less sharp-edged structure with nanodots by Al doping.

**Figure 4:**
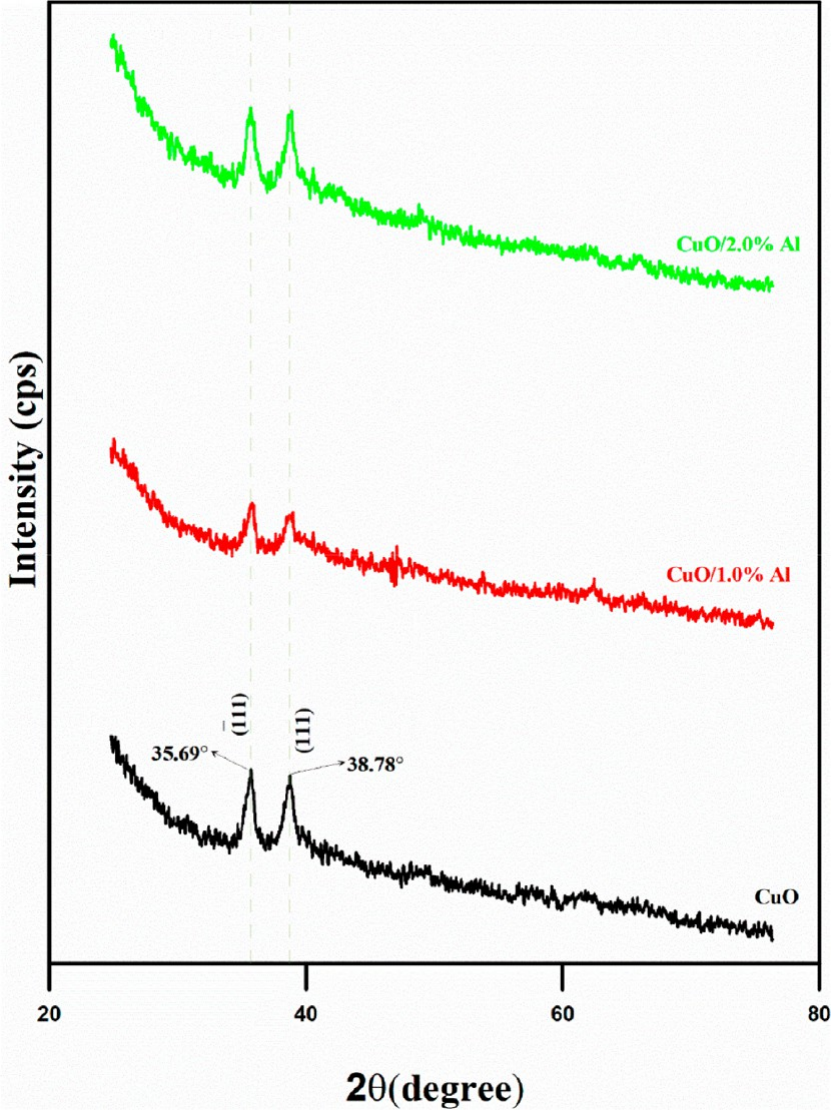
X-ray diffraction patterns of flexible CuO films with Al content ranging from 0.0 to 2.0%. Increasing the Al content leads first to a decrease and then to an increase in the peak intensity of the flexible films. This causes a change in the lattice symmetry.

The XRD procedure is one of the important methods to study the structural characteristics of the produced nanomaterials, such as crystal structure, phase purity, and crystallinity. The XRD spectra of the synthesized CuO, CuO/1.0% Al, and CuO/2.0% Al films were recorded in the 2θ = 20°–80° range as given in Figure 4. Bare and Al-doped CuO films with different contents give two different dominant diffraction peaks at 35.69° and 38.78° corresponding to (1̅11) and (111) crystallographic plane reflections, respectively (compatible with 00-041-0254 JCPDS file number).^21^ No Al-related secondary phase and impurity peaks were detected, indicating that the doping of different percentages of Al^3+^ ions does not change the crystal symmetric structure of the CuO material. This means that the Al element had no effect on the crystal structure of the host CuO, so the monoclinic crystal structure of CuO was preserved in all films. As shown in Figure 4, it is seen that the Al doping content affects the diffraction peak intensities of the films. With the addition of 1% Al to the reaction solution, the peak intensity of the films first decreased (from 109 to 55.4 cps for the (1̅11) plane) and then the peak intensity of the films increased (from 55.4 to 104 cps for the (1̅11) plane) with the addition of 2.0% Al. The change in the peak heights of the films may be due to vacancies and defects formed by the addition of Al^3+^ to the lattice. These defects and vacancies affect the symmetry of the crystal lattice, resulting in charge imbalance.^22^

The average crystallite size (*D*) values for planes (1̅11) and (111), where λ is the X-ray wavelength (1.5406 Å, Cu Kα radiation), β is the full width at half-maximum, and θ is the Bragg’s diffraction angle, were calculated using Debye–Scherrer’s relation.^23^
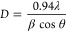
1*D* values for bare CuO, CuO/1.0% Al, and CuO/2.0% Al films were calculated as 13.20, 8.32, and 17.18 nm, respectively. This change in *D* values may be due to the shift and broadening of the peaks due to defects in crystal symmetry caused by the addition of Al^3+^ to the solution. This difference can also be attributed to differences in the radii of Cu^2+^ (0.57 Å) and Al^3+^ (0.39 Å) (for a 4-coordination number).^24^

**Figure 5:**
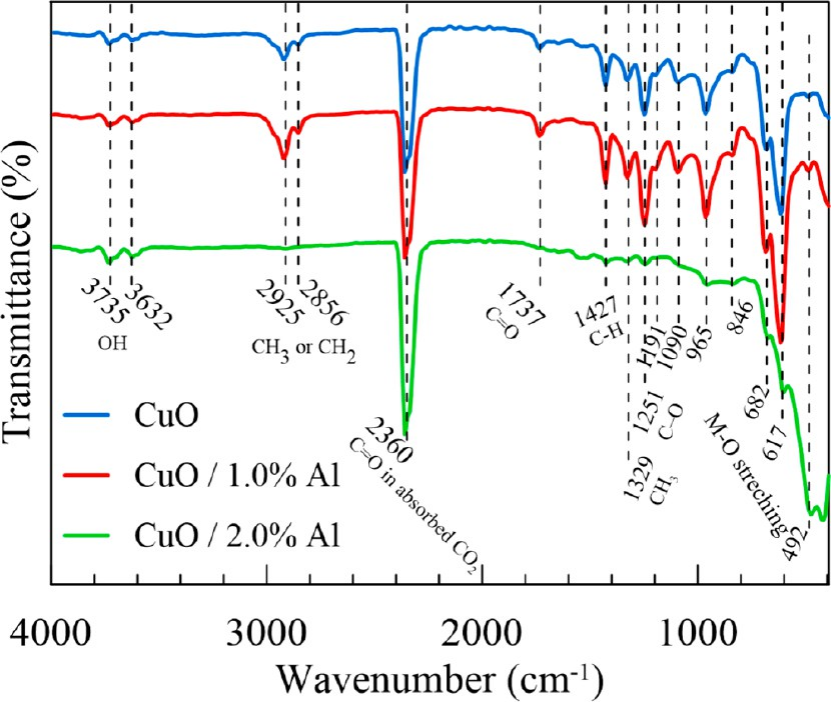
FTIR spectra of undoped and 1.0% and 2.0% Al-doped CuO films.

Figure 5 displays the FTIR spectra of bare and 1.0% and 2.0% Al-doped CuO films grown on cellulose acetate (CA) substrates by using the SILAR method, Figure 5. CuO and similar metal oxide materials exhibit unique absorption bands below 1000 cm^–1^ (fingerprint region), resulting from strong stretching vibrations. The FT-IR spectrum of undoped CuO has four typical intense peaks incorporated with the Cu–O vibrations of monoclinic CuO at 420 cm^–1^, 492, 617, and 846 cm^–1^.^25^ These absorption peaks slightly shifted to lower values due to the decrease in the surface area and surface defects with Al doping.^25a,25c,25d,26^ Also, in the rest of the FTIR spectra, strong absorption peaks of cellulose acetate were observed in all spectra because of the penetration depth of IR.^25a^ In particular, the bare and 1.0% and Al-doped CuO film spectra show three major peaks of the acetyl group (in CA).

The CA characteristic peak of C–O–C at 1050 cm^–1^ in anhydroglucose units not observed but three important acetyl group vibrations at 1737 cm^–1^ (υ_C=O_), 1329 cm^–1^ (υ_–CH3_), and 1251 cm^–1^ (υ_C–O_) exist in the spectra.^25b,27^ Because of the acetylation process, the peaks of −OH functional groups and absorbed water molecules were reduced.^25a^ In addition, bare and Al-doped CuO on CA has an absorption band at 2360 cm^–1^ due to the stretching vibration of CO_2_ molecules adsorbed from air.^25a,28^ In addition to all these, a significant increase was observed in the peaks of the flexible CA substrate due to the decrease in the thickness of the 2.0% Al-doped CuO films.

We characterized the sweat loss tracking performance of the fabricated flexible devices by providing four different concentrations of artificial sweat samples that were dropped onto the devices. The sensing-response values are defined as eq 2(18)
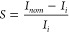
2where *S* is the sensing response value of the CuO thin film material to the artificial sweat solutions. *I*_*nom*_ is the stable conductivity of the devices in the sweat solution implementation; *I*_*i*_ is the steady conductivity of the device in air (without artificial sweat solution implementation).

**Figure 6:**
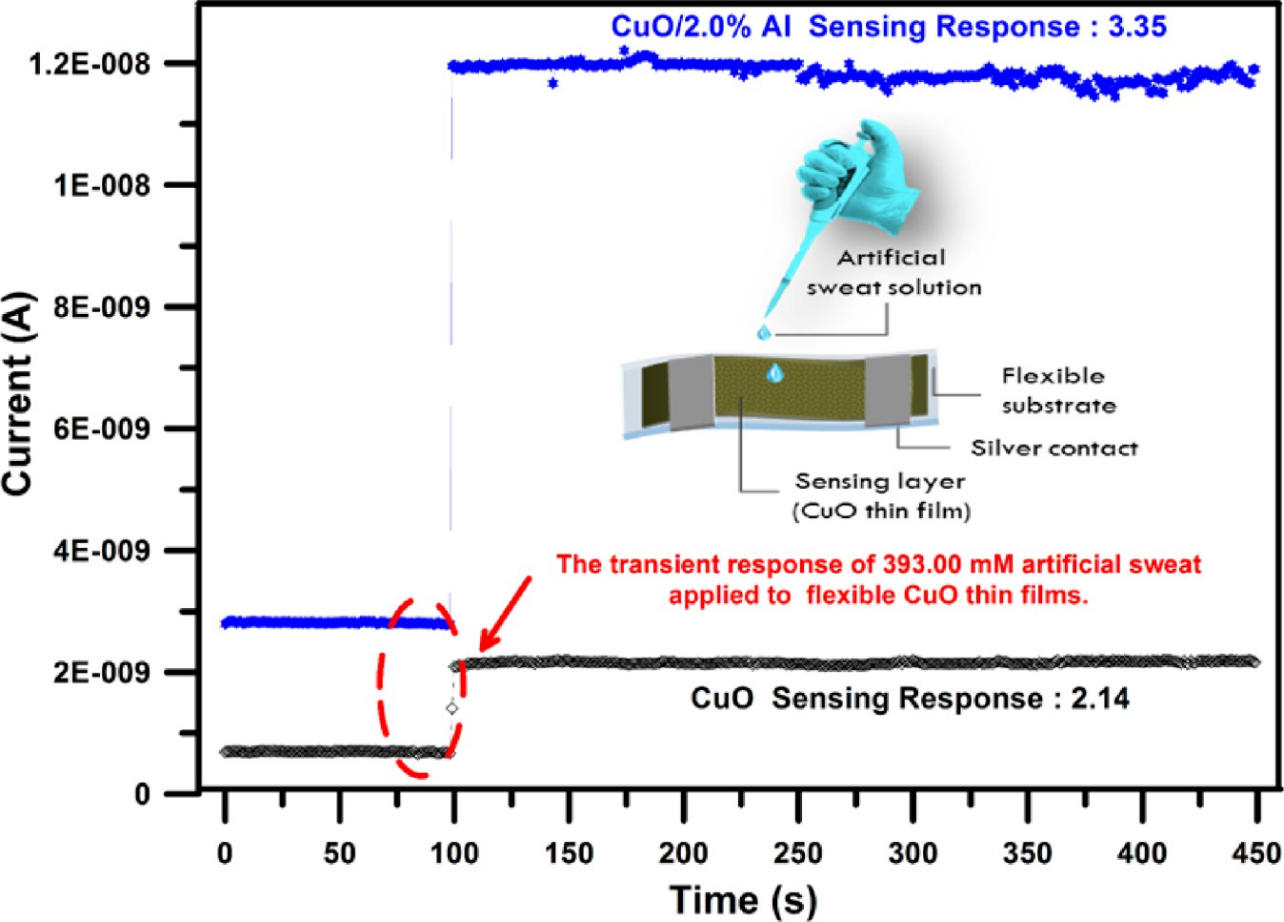
To provide practical application, we investigated the working efficiency of Al:CuO at room conditions since most clinical and healthcare industries operate under ambient temperature conditions. It can be seen from Figure 6 that fabricated flexible devices immediately respond to the implementations of sweat-solution and reach a steady-state value for a short time.

Figure 6 reveals the corresponding sensing response performances of the two sensors (bare and 2.0% Al-doped CuO) toward 393.00 mM sweat solution at room temperature. To provide a practical application, we investigated the working efficiency of Al:CuO under room conditions since most clinical and healthcare industries operate under ambient temperature. It can be seen from Figure 6 that the fabricated flexible devices immediately respond to the implementations of sweat solutions and reach a steady-state value within a short time. Note that the bare CuO film had partially lower sensing response values than the 2.0% Al:CuO sample for the same sweat solution concentration. Despite the undoped sample being responsive to 393.00 mM sweat-solution (S = 2.14), the sensing response reaches 3.35 for the 2.0% Al-doped CuO device. In addition, the magnitude of the conductivity obtained for the Al-doped CuO sample was found to be larger as compared with the bare CuO sample. The enhanced sensing ability of CuO:Al nanostructures is chiefly attributed to the morphological evolution of undoped CuO structures. The alteration of the surface morphology of the CuO structure with Al-doping ensures more adsorption of implemented solutions and therefore higher responses are achieved. As can be observed in FESEM images and contact angle measurements, the size, distribution, and shape of particles changed with Al-doping and the contact angle decreased which leads to a boost of sweat-solution interaction with the device structure. The modification of the surface properties of the CuO structure with Al-doping ensures more active sites for the adsorption of implemented solutions and therefore higher responses are achieved. Meanwhile, the Al-donor dopant for CuO increases the hole accumulation layer width and hence could enhance the sensing response.^13a,29^ The microstructural alteration caused the formation of heterogeneous center grain boundaries of Al-CuO. The changed barrier height of Al-doped CuO may be responsible for the decreasing resistance of samples.^30^ This surveillance was similarly acquired in other Al-substituted CuO thin film research and was attached with the reason for the altering carrier concentrations in the device leading to the advancement in mobility.^14,31^

**Figure 7:**
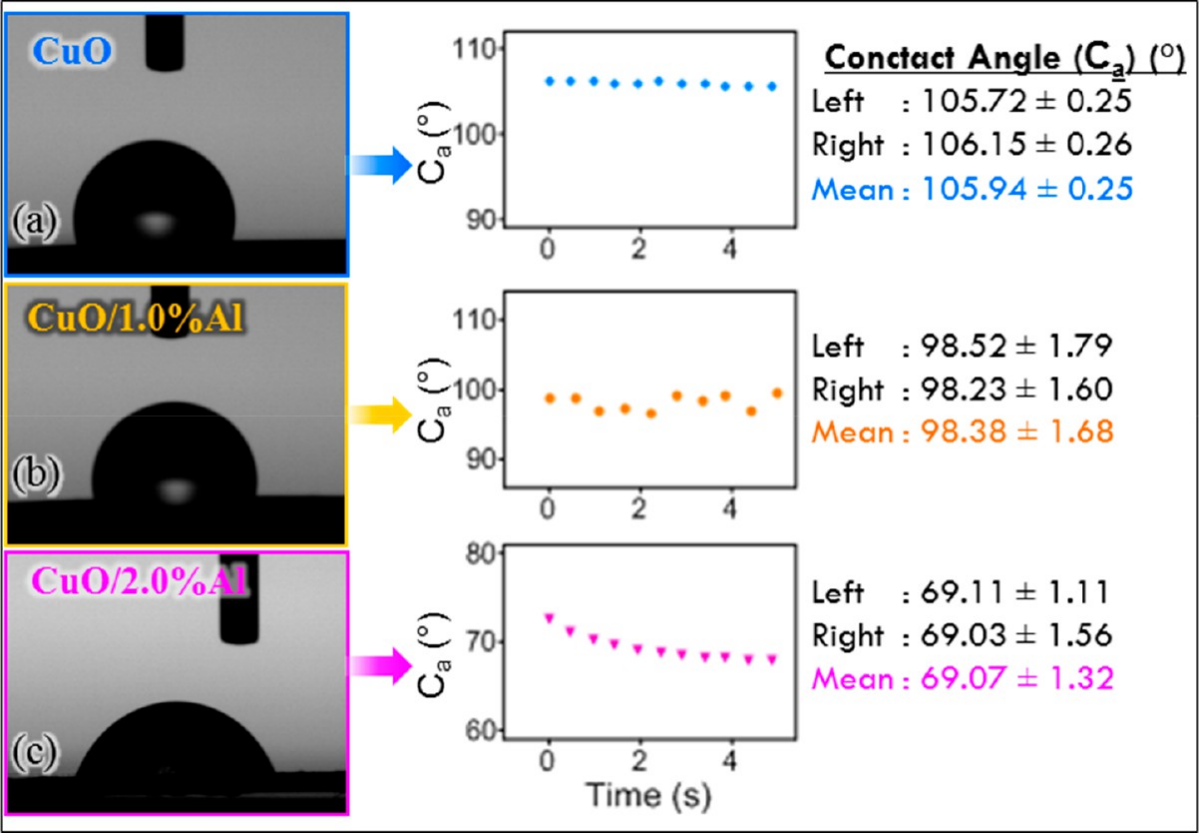
Artificial sweat contact angles on different surfaces: (a) bare CuO thin film, (b) CuO/1.0% Al thin film, and (c) CuO/2.0% Al thin film. The static contact angle for the bare CuO sample was found to be 105.94°. However, after the Al-doping of the CuO samples, the surface was found to be hydrophilic with a contact angle of 69.07° for the CuO/2.0% Al sample which leads to a boost of sweat-solution interaction with the device structure.

Figure 7 presents pictures of artificial-sweat drops on the surface of CuO thin films with different Al concentrations, exhibiting the decreasing contact angle hydrophilic nature of the CuO samples. The static contact angle for the bare CuO sample was found to be 105.94°. However, after Al-doping of the CuO samples, the surface was found to be hydrophilic with a contact angle of 69.07° for the CuO/2.0% Al sample, which leads to a boost of sweat-solution interaction with the device structure. This increased interaction between the artificial-sweat sample and the designed device surface is potent for the sensing system and causes a significant sensing characteristic. Inhalation of severe sweat-electrolytes in the sensing systems is important to enhance the active sites on the device structure and improve sensing performances.^18,32^ It indicates that the enhanced sweat-sensing response for the changed contact angle is due to the adsorption of more ions on the film surface.

**Figure 8:**
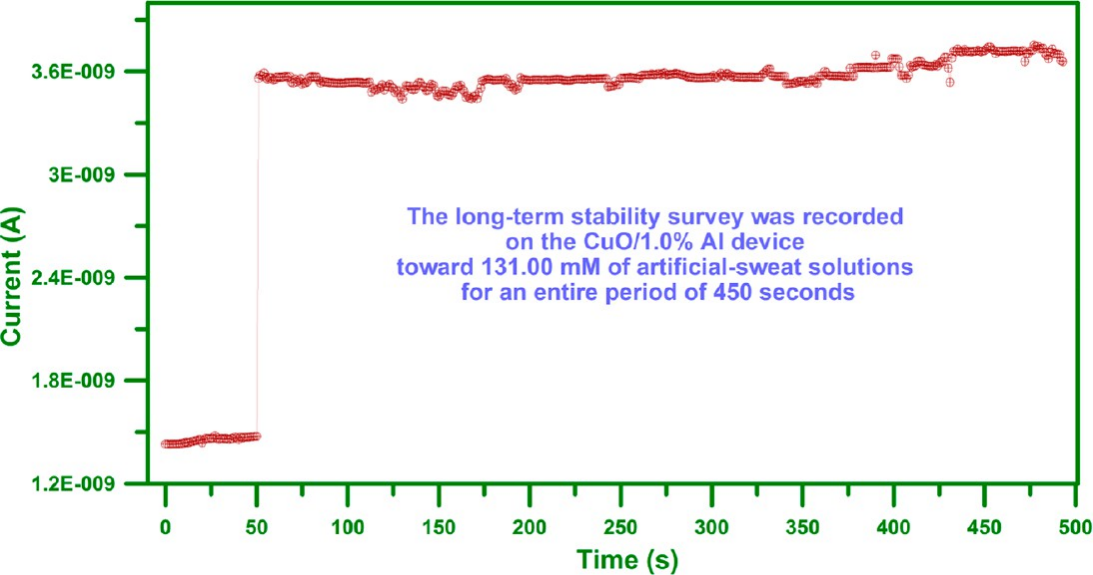
The stability analysis was performed on the CuO/1.0% Al sample toward 131.00 mM of sweat-solution for a total period of 450 s, as presented in this figure. This is evident that the CuO/1.0% Al device presents decent stability for the dense electrolyte content of the artificial-sweat application.

During the real-time-tracking procedure, sensing response performance not only implies an enhanced response but also implies long-term stability. For a sensing system, stability deals with the degree to which sensor characteristics remain constant over time. In this manner, stability analysis was performed on the CuO/1.0% Al sample toward 131.00 mM of sweat solution for a total period of 450 s, as presented in Figure 8. This is evident that the CuO/1.0% Al device presents decent stability for the dense electrolyte content of the artificial-sweat application. Owing to further evaporation and absorption of the implemented solution, the current value fluctuates within the 5.0% range.

Moreover, the sensing-response efficacy of fabricated samples was examined for a relatively long period, namely, for 4 weeks at room conditions, to record its required functions under stated conditions for a specified period and the reusability of designed systems. The artificial sweat sensing efficacy was diminished by about 10% after 1 month. The possible reason for this small reduction in sensing performance after 4 weeks is mainly due to the second enforcement of the identical sweat ingredient on the thin film could be leading to a low interplay between the electrolytes and the sample surface.^33^

The limits of detection (LOD) amounts of the fabricated sensors were determined using the calibration graph (current versus artificial sweat concentration).^34^ The undoped sample was determined to exhibit a low range of the LOD (3.90 mM), and a remarkable decrease in the LOD was observed with Al-doping of 3.65 and 3.40 mM for 1.0 and 2.0% CuO/Al devices, respectively.

**Figure 9:**
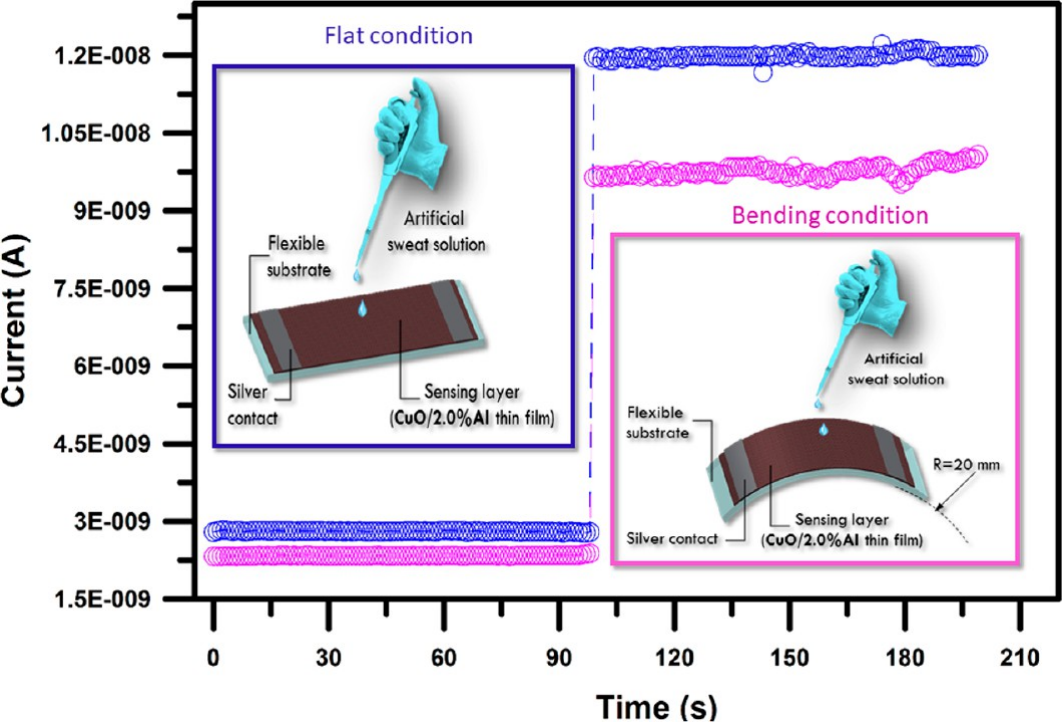
Transient responses of the flexible CuO/2.0% Al thin films to a proportionally dense electrolyte content of sweat (393.00 mM). It was observed that the fabricated CuO/Al systems are capable of sensing artificial sweat under 40 mm bending conditions. But, the response performances of the samples are to some degree influenced due to bending.

For wearable electronics, the mechanical flexibility of the fabricated materials is important. In order to understand the sensing performances of the CuO/Al samples under bending conditions, the designed materials were attached to the surface of the cylinder rod with radii of 40 mm under room conditions. It was observed that the fabricated CuO/Al systems are capable of sensing artificial sweat under 40 mm bending condition. But the response performances of the samples are to some degree influenced due to bending. A reduction of ∼11.00% and ∼13.00% was recorded due to landings of devices for the bare CuO and 2.0% Al-doped samples, respectively (Figure 9). Because nanoscale fabricated samples are harsh, fragile cracks came about during the bending treatment. The resistance of the nanoscale thin film materials depends on the strain in the structure, which for bending is a function of the bending radius. As has been recorded by Torrisi et al.,^35^ a thin film under bending stress will have a higher tendency to decay via cracks occurring in the sample structure. Also, thin film samples that have large grain sizes may be easily delaminated from the substrate under bending conditions, giving an increase in conductivity deterioration. In general, the resistivity of p-type oxide materials increases with respect to the strain due to reduced mobility due to structural deterioration.^36^

**Figure 10:**
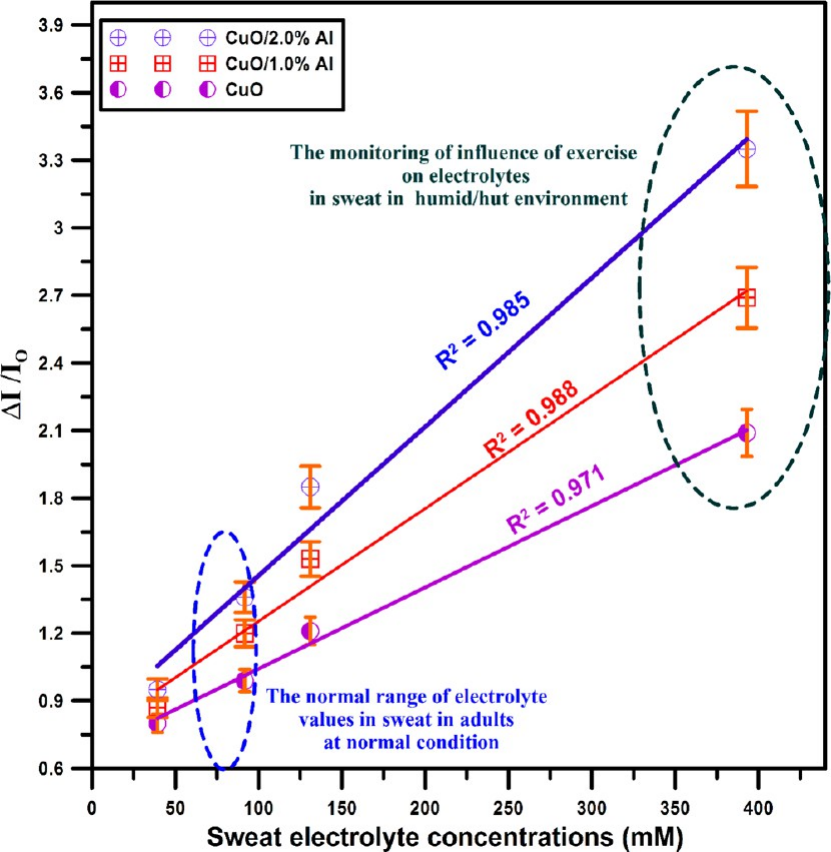
It is concluded from this figure that the value of the sweat sensing response increases with an increasing concentration of Al in the bath solution. Also, response ratios of the devices have a linear dependence on the Al content in the growth bath which implies that aluminum as a dopant element could be used as a regulator for the important physical characteristics of metal-oxide materials.

Fabricated CuO/Al-based thin film materials were performed for four different concentrations of artificial sweat, and the sensing response values are plotted in Figure 10. It is concluded from Figure 10 that the value of the sweat sensing response increases with the increasing concentration of Al in the bath solution. Also, the response ratios of the devices have a linear dependence on the Al content in the growth solution, which implies that aluminum as a dopant element could be used as a compensator for the important characteristics of metal-oxide structures. To further clarify the enhanced sensing characteristics of Al-doped samples toward sweat solution, two main agents are accountable: (I) decreased Sa and Sq parameters influence the contact angle of the implemented solution on the surface, causing an enhancement in the sensing performance, and (II) superior surface-to-volume ratio as a consequence of nanoscale structure leads to higher absorption of the sweat. Meantime, the sweat sensing contraption of the flexible CuO systems comprises the switch in the surface resistance due to the chemisorptions of sweat ions on the surface of the sensor. The sweat solution involves ions mainly from potassium (K) and sodium (Na), and more adsorption of the ions on the sample surface is very important to improve the active sites on the surface sensor and enhance sensing efficacies.^32,37^

Figure 10 additionally presents the linear fit of the designed material response efficacy due to the artificial sweat solution concentration. All fabricated thin film based devices present a linear response for the four concepts of an artificial sweat solution. The 2.0% Al-doped CuO-based device shows a higher response value than that of the remaining devices. This considerable improvement in response performance is most commonly a result of the alteration in electron exchange at the device surface due to the dopant substitution.^38^ The artificial-sweat sensing performances of flexible nanoscale bare CuO and Al:CuO sensors were exhibited in this study. Al-doping CuO can realize real-time tracking of sweat loss at room temperature with easy conductometric measurements that do not require complicated fabrication and instrumentation processes. This nanoscale flexible CuO thin film based device structure will lead to a new approach for further research of flexible thin film based sensing material.

## 4 Conclusions

We proposed to develop a real-time sweat-loss tracking system with nanoscale flexible CuO/Al thin film-based equipment in hot and humid climates. Therefore, nanostructured flexible CuO films with different Al element percentages were synthesized on a cellulose acetate substrate by using the SILAR method. From the FE-SEM images, it is seen that the flat plate flower-like patterned CuO structures are transformed into less sharp-edged structures containing nanodots with Al doping. Similarly, it is also clear from the AFM images that the surface shape of films changes with the incorporation of Al. It is observed that all CuO films obtained from XRD analyses crystallize in a polycrystalline structure. Furthermore, XRD diffraction patterns show that the crystallite sizes and peak heights change with the addition of Al to the solution. It was found from the wettability results that the surface of the CuO films after Al doping was hydrophilic, with a contact angle of 69.07° for the CuO/2.0% Al sample. When all the samples produced are compared, it is concluded that the value of sweat sensing response increases as the percentage of Al in the solution increases, i.e., CuO/2.0% Al film has a better sweat sensing response. The bare and 1.0% and 2.0% Al substituted CuO films provide excellent linearity with linear regression ranges (*R*^2^) of 0.971, 0.988, and 0.985, respectively. From the results obtained, it is expected that Al-doped flexible CuO films can be used in sweat-level monitoring, which is a biological fluid, depending on sweating. We also hope that these flexible films can be developed as alternative sweat sensors, as they are reproducible and inexpensive to produce.
